# Linking Cardiac and Renal Hemodynamics for the Prevention of Contrast-Induced Nephropathy

**DOI:** 10.1016/j.jscai.2026.105520

**Published:** 2026-08-04

**Authors:** Kevin Haddad, Margaret McEntegart, Ajay J. Kirtane, Megha Prasad

**Affiliations:** Department of Medicine, NewYork-Presbyterian Hospital/Columbia University Irving Medical Center, New York, New York

**Keywords:** contrast-induced acute kidney injury, left ventricular end-diastolic pressure, renal hemodynamics

Acute kidney injury following contrast exposure (contrast-induced acute kidney injury [CI-AKI]) is associated with increased morbidity and mortality, including higher rates of myocardial infarction, stroke, progression to chronic kidney disease, and death.^1^ Although CI-AKI risk can be mitigated through strategies such as minimizing contrast volume and optimizing periprocedural hemodynamics, these interventions remain variably applied in clinical practice. A clear understanding of renal hemodynamics is therefore essential to guide physiologically sound strategies aimed at optimizing hemodynamics and reducing the risk of CI-AKI.

## Renal hemodynamics

Renal hemodynamics depend on tightly regulated renal blood flow (RBF) and glomerular filtration rate (GFR), governed by both intrinsic renal autoregulatory mechanisms and systemic influences. A central determinant of renal perfusion is renal perfusion pressure (RPP), defined as the gradient between mean arterial pressure (MAP) and renal venous pressure (RVP) as follows^2^:

(1) Equation 1: RPP=MAP−RVP

This concept highlights the importance of venous backpressure—not only hypotension—as a determinant of renal dysfunction in the catheterization laboratory.

At the glomerular level, GFR is governed by Starling forces across the filtration barrier, including glomerular capillary hydrostatic pressure (approximated by MAP), Bowman space (BS) hydrostatic pressure, and oncotic pressure gradients (π).^2^ Increases in MAP generally favor filtration, whereas elevations in BS pressure or plasma oncotic pressure oppose it as follows:

(2) $$\text{Equation 2: GFR}\propto \left(P_{\text{GC}}-P_{\text{BS}}\right)-\left(\pi_{\text{GC}}-\pi_{\text{BS}}\right)$$

To preserve RBF across a wide range of perfusion pressures, the kidney relies on 2 primary intrinsic autoregulatory mechanisms. The myogenic response enables afferent arteriolar smooth muscle to constrict in response to increased transmural pressure, stabilizing flow via stretch-activated calcium signaling.^3^ Complementing this mechanism, tubuloglomerular feedback adjusts afferent arteriolar tone in response to distal tubular sodium chloride delivery. These finely tuned responses are further modulated by the renin–angiotensin–aldosterone system, nitric oxide, reactive oxygen species, and extrinsic inputs such as the sympathetic nervous system and natriuretic peptides.^4^ Importantly, efferent arteriole vasoconstriction, mediated predominantly by angiotensin II, helps preserve glomerular capillary pressure and GFR during reductions in renal perfusion.

## Contrast effect on renal hemodynamics

Against this tightly regulated physiologic background, iodinated contrast administration induces notable perturbations in renal hemodynamics. Contrast exposure produces a biphasic vascular response—an initial transient vasodilation, followed by sustained vasoconstriction—resulting in reduced RBF and GFR.^4^ These effects are magnified by higher contrast and viscosity.^4^ Concurrently, increased distal sodium delivery due to contrast activates tubuloglomerular feedback, further promoting afferent vasoconstriction, particularly in patients with chronic kidney disease or metabolic derangements, in whom autoregulatory reserve is already impaired.^4^ Chemotoxic pathways play a central role, characterized by increased endothelin and adenosine release with concomitant reductions in nitric oxide and prostaglandin synthesis, collectively promoting renal vasoconstriction. The hemodynamic effects of contrast media are thought to occur primarily at the afferent arteriole, although changes in efferent arteriolar tone may also influence intraglomerular pressure.^5^

Beyond their vasoreactive properties, iodinated contrast agents exert direct cytotoxic effects on renal tubular epithelial cells. These alterations lead to a mismatch between oxygen delivery (DO_2_) and oxygen consumption (VO_2_), with impaired DO_2_ driven by increased blood viscosity and heightened VO_2_, resulting from enhanced tubular sodium transport activity. The resulting imbalance predisposes the renal medulla to hypoxia, culminating in tubular cell injury and necrosis.

## Translating pathophysiology into clinical treatment pearls

**Figure 1:**
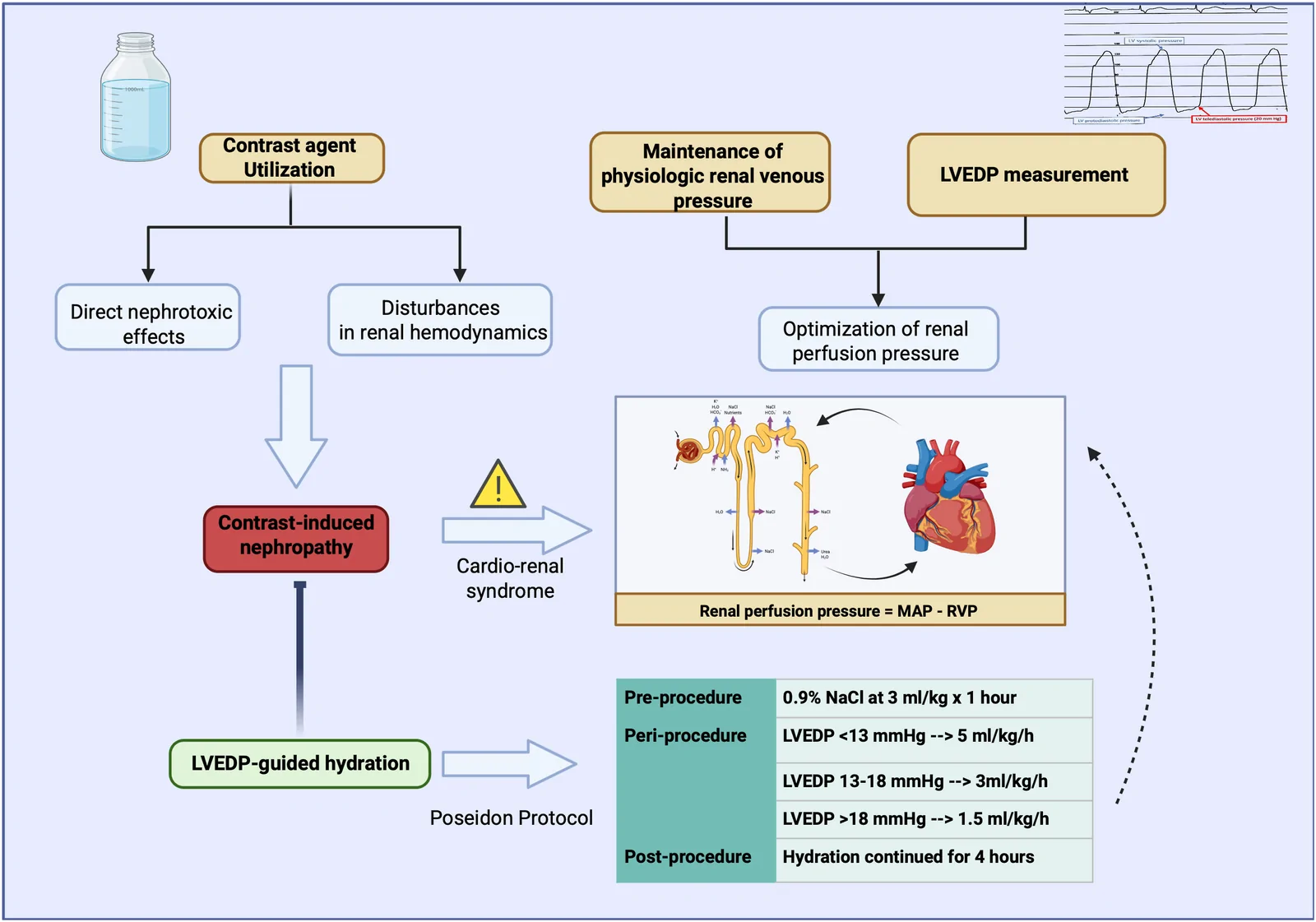
**Integrating cardiac filling pressures and renal hemodynamics to mitigate contrast-induced kidney injury.** Iodinated contrast agents cause kidney injury through direct tubular toxicity and renal hemodynamic disturbances. Inadequate volume expansion and elevated renal venous pressure reduce renal perfusion pressure, worsening cardiorenal interactions. Left ventricular end-diastolic pressure (LVEDP)-guided hydration, as implemented in the POSEIDON protocol, individualizes isotonic saline administration to optimize renal perfusion and reduce contrast-related renal toxicity. MAP, mean arterial pressure; RVP, renal venous pressure. Created in BioRender. Haddad, K. (2026) https://BioRender.com/mztgp71.

Effective prevention of CI-AKI begins with systematic risk stratification using validated tools such as the Mehran score, with baseline GFR and diabetes among the strongest predictors.^6^ Avoiding nephrotoxic drugs and periprocedural volume expansion remain the cornerstone of prevention (Figure 1). Optimizing hemodynamics to maintain adequate MAP while minimizing venous congestion helps preserve RPP and GFR (Figure 1). As demonstrated in the POSEIDON trial, left ventricular end-diastolic pressure (LVEDP)-guided hydration individualizes fluid administration using LVEDP as a marker of volume tolerance, allowing sufficient volume expansion to enhance renal perfusion and urine output while minimizing the risk of volume overload and, in certain clinical settings, subsequent venous congestion.^7^ Although diuretics and vasodilators may reduce RVP in selected patients, hydration remains the cornerstone of CI-AKI prevention because it provides intravascular volume expansion, which both supports renal perfusion and reduces the nephrotoxic effects of contrast media through dilution and enhanced urinary flow.

The protocol consisted of preprocedural infusion of 0.9% NaCl at 3 mL/kg for 1 hour, followed by LVEDP-guided postprocedural infusion for 4 hours: 5 mL/kg/h (LVEDP < 13 mm Hg), 3 mL/kg/h (LVEDP = 13-18 mm Hg), and 1.5 mL/kg/h (LVEDP > 18 mm Hg) (Figure 1).^7^ LVEDP-guided hydration significantly reduced contrast-associated acute kidney injury by ∼59% compared with standard hydration in patients undergoing cardiac catheterization (6.7% vs 16.3%; relative risk, 0.41; 95% CI, 0.22-0.79; *P* = .005). Adherence to best clinical practices aimed at minimizing the risk of CI-AKI should also include the adoption of ultralow contrast strategies, with efforts to maintain the total contrast volume at or below the estimated GFR whenever feasible.

## Conclusion

Adequate volume optimization remains the most effective strategy to reduce CI-AKI risk. LVEDP provides a practical, intraprocedural surrogate of venous congestion and preload, enabling physiology-guided hydration that balances RPP and RVP. This hemodynamically informed approach offers a coherent, mechanism-based framework for contrast nephropathy prevention in the catheterization laboratory.

**Pearls in Hemodynamics**

• Renal perfusion pressure is the difference between mean arterial pressure and renal venous pressure; both hypotension and elevated venous pressure can impair kidney function.
• Contrast media disrupt renal autoregulation, leading to reduced renal blood flow, medullary hypoxia, and tubular injury.
• Adequate intravascular volume expansion remains the most effective strategy to reduce contrast-induced acute kidney injury risk.
• Left ventricular end-diastolic pressure–guided hydration allows tailored fluid administration based on preload status and has been shown to reduce contrast-induced acute kidney injury compared with standard hydration.

## CRediT authorship contribution statement

**Kevin Haddad:** Writing – original draft. **Margaret McEntegart:** Writing – review & editing. **Ajay J. Kirtane:** Writing – review & editing. **Megha Prasad:** Writing – review & editing, Supervision.

## Declaration of competing interest

Ajay J. Kirtane reports institutional funding to Columbia University and/or Cardiovascular Research Foundation from Abbott Vascular, Amgen, Bolt Medical, Boston Scientific, CathWorks, Concept Medical, Cordis, Idorsia, Janssen, Magenta Medical, Medtronic, Neurotronic, Philips, ReCor Medical, Supira, and Teleflex. In addition to research grants, institutional funding includes fees paid to Columbia University and/or Cardiovascular Research Foundation for consulting and/or speaking engagements in which Ajay J. Kirtane controlled the content. Ajay J. Kirtane reports other personal interests as follows—equity options in Airiver, Aeroflow, Bolt Medical, Lumen Medical, Roon, and Tonic Medical; consulting fees from Sonivie; travel expenses/meals from Abbott Vascular, Acist, Abiomed, Amgen, AngioDynamics, Biotronik, Boston Scientific, CathWorks, Chiesi, Concept Medical, Edwards Lifesciences, Kiniksa Pharmaceuticals, Medtronic, Novartis, Janssen, Philips, ReCor Medical, Shockwave Medical, and Supira. Kevin Haddad, Margaret McEntegart, and Megha Prasad declared no potential conflicts of interest with respect to the research, authorship, and/or publication of this article.
